# Establishment of a peptide-based enzyme-linked immunosorbent assay for detecting antibodies against PRRSV M protein

**DOI:** 10.1186/s12917-021-03060-z

**Published:** 2021-11-19

**Authors:** Jun Zhao, Rubo Zhang, Ling Zhu, Huidan Deng, Fengqing Li, Lei Xu, Jianbo Huan, Xiangang Sun, Zhiwen Xu

**Affiliations:** 1 College of Veterinary Medicine, Sichuan Agricultural University, Chengdu, 611130 China; 2 Key Laboratory of Animal Diseases and Human Health of Sichuan Province, Chengdu, 611130 China; 3 College of Animal Science, Xichang University, Xichang, 615000 Sichuan China

**Keywords:** Porcine reproductive and respiratory syndrome virus, M protein, Synthetic peptide, Enzyme-linked immunosorbent assay, NADC30-like PRRSV inactivated vaccine, Pig

## Abstract

**Background:**

Porcine reproductive and respiratory syndrome (PRRS) is one of the most economically devastating diseases affecting the swine industry globally. Evaluation of antibody responses and neutralizing antibody titers is the most effective method for vaccine evaluation. In this study, the B cell line epitopes of PRRSV M protein were predicted, and two peptide ELISA assays were established (M-A110-129 ELISA, M-A148-174 ELISA) to detect antibodies against PRRSV M protein. Field serum samples collected from pig farms were used to validate the peptide ELISA and compare it with an indirect immunofluorescence assay.

**Results:**

The sensitivity and specificity of M-A110-129 ELISA and M-A148-174 ELISA were (111/125) 88.80%, (69/70) 98.57% and (122/125) 97.60%, (70/70) 100%, relative to indirect immunofluorescence assay. This peptide ELISA could detect antibodies against different genotypes of PRRSV including type 1 PRRSV, classical PRRSV, HP-PRRSV, and NADC30 like PRRSV, but not antibodies against other common swine viruses. The results of ROC analysis showed that the area under the curve (AUC) of the M-A110-129 ELISA and M-A148-174 ELISA were 0.967 and 0.996, respectively. Compared the concordance of results using two peptide ELISA assays, the IDEXX PRRSV X3 Ab ELISA and a virus neutralization test, were assessed using a series of 147 sera from pigs vaccinated with the NADC30-like PRRSV inactivated vaccine. The M-A148-174 ELISA had the best consistency, with a Cohen’s kappa coefficient of 0.8772. The concordance rates of the Hipra PRRSV ELISA kit, M-A110-129 ELISA and M-A148-174 ELISA in the field seropositive detection results were 91.08, 86.32 and 95.35%, relative to indirect immunofluorescence assay.

**Conclusions:**

In summary, compared with M-A110-129 ELISA, the PRRSV M-A148-174 ELISA is of value for detecting antibodies against PRRSV and the evaluation of the NADC30-like PRRSV inactivated vaccine, but the advantage is insufficient in serological early diagnosis.

**Supplementary Information:**

The online version contains supplementary material available at 10.1186/s12917-021-03060-z.

## Background

Porcine reproductive and respiratory syndrome virus (PRRSV) is the causative agent of porcine reproductive and respiratory syndrome (PRRS). It is an economically devastating pandemic disease of swine that mainly infect the respiratory system and reproductive system [1]. PRRSV infections cause abortion, stillbirth and mummification in gestation sows and respiratory disorders in nursery pigs [2]. PRRSV is a small enveloped, single-stranded, positive-sense RNA virus [3]. The viral genome is approximately 15 kb. PRRSV encodes at least 10 open reading frames (ORFs), comprised of ORF1a, ORF1b, ORF2a, ORF2b, ORFs3-7 [4, 5]. ORF1a and ORF1b encode viral replicase polyproteins, which are hydrolyzed into 16 mature non-structural proteins by the viral protease [6]. ORFs3-7 encode viral GP2-5, M, E and the nucleocapsid protein N [7]. The N protein of PRRSV has the highest immunogenicity among all structural proteins, but the N protein antibody produced by the host does not neutralize the virus. Antibodies produced by the host in the early stage of PRRSV infection are mainly against the N protein, and antibodies against the N protein can be detected 1 week after infection [8]. In pigs naturally infected with PRRSV, neutralizing antibodies are produced later and the neutralizing titer is low [9]. The main neutralizing epitopes of PRRSV are mainly distributed in the GP5 protein, which shows considerable variability among different subtypes and strains [10, 11]. The M protein is a non-glycosylated membrane matrix protein encoded by ORF6. It has strong immunogenicity and contains a large number of antigenic epitopes [12, 13]. The M protein is also the most conservative protein in PRRSV structural proteins. Specific antibodies against the M protein can be detected in the early stage of virus infection, and the antibody level remains stable for a long time [14].

There are some controversies about PRRSV vaccination, but vaccination is still one of the most important assays for epidemic prevention and control. Antibody determination is the most effective method to evaluate the immune effect of a vaccine. Currently, The IDEXX PRRSV X3 Ab ELISA kit is the most commonly used commercial kit for PRRSV antibody detection because of its high sensitivity and effectiveness. The IDEXX PRRSV X3 Ab ELISA utilizes plates coated with the viral N protein. PRRSV induces anti-N protein antibodies early, at a large scale and stably after infecting the host. However, commercial kits are expensive, which is an important limitation. Therefore, it is necessary to develop a stable, rapid, accurate and inexpensive PRRS antibody detection method. Synthetic peptide antigens have the advantages of good stability, standardization and high consistency. There have been many successful cases of using synthetic peptide antigens in ELISA. At present, SARS-CoV-2, getah virus (GETV), infectious bronchitis virus (IBV), avian infectious laryngotracheitis virus (ILT), zika virus, influenza A viruses have been established ELISA antibody detection assays based on synthetic peptide antigens [15–19]. Several ELISA assays based on peptide have been established and evaluated for PRRSV of European and American types. The peptides used for encapsulation are located in Nsp2, Gp4, GP5, M and N proteins [20–24].

In our study, two peptides based on the previous epitope research and bioinformatics analysis were selected for the evaluation of specificity and sensitivity of two peptide ELISA assays for anti-PRRSV antibody detection using serum samples that were either negative or positive for anti-PRRSV antibodies. The antibody production of pigs immunized with the NADC30-like PRRSV autogenous inactivated vaccine was evaluated by the established peptide ELISA antibody detection assays, the IDEXX PRRSV X3 Ab ELISA kit and a virus neutralization test to analyze the concordance between the virus neutralization test, IDEXX PRRSV X3 Ab ELISA, and peptide ELISA. Comparison the detection results of peptide ELISA, IDEXX PRRSV X3 Ab ELISA kit, Hipra PRRSV ELISA kit and IFA for PRRSV specific antibodies in field serum samples.

## Results

### PRRSV M protein analysis and B cell line epitopes prediction

**Fig. 1:**
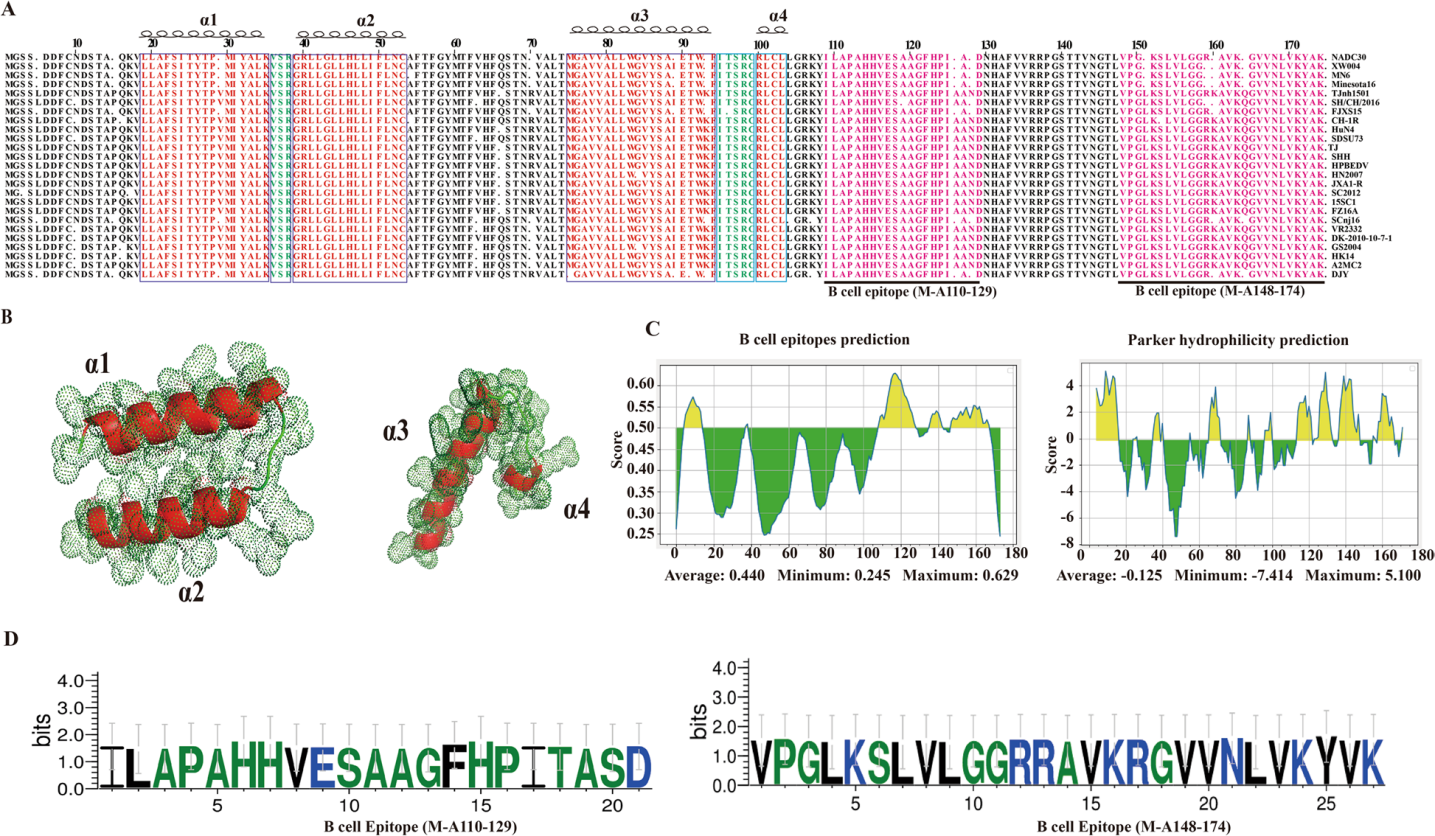
Amino acid alignment, secondary structure prediction and B cell linear epitope prediction of PRRSV M protein. **A** Amino acid alignment of PRRSV M protein. The alpha helix regions are marked in red, and irregular curls are marked in green. The B cell epitopes selected in this study are marked in purple. **B** The 3D pattern of the secondary structure predicted in PRRSV M protein. **C** Prediction of B cell linear epitopes and hydrophilicity in PRRSV M protein by IEDB. **D** Peptide sequences used in this study

The M protein amino acid of HP-PRRSV and NADC30-like PRRSV strains sequence alignment showed that aa5 L-I, aa10 H-N, aa15 P-V, aa29 V-I, aa63 A-V, aa66 E-Q, aa70 R-K, aa89 I-M, aa93 K-R, aa126 A-T, aa128 N-S, aa151 L-F, aa159 R-S, aa160 K-R and aa164 Q-R were replaced (Fig. 1A). Secondary structure simulation results showed that PRRSV M protein contained four α-helix structures (aa19-35, aa39-53, aa75-94, aa99-103) and two irregular coils (aa36-38, aa95-98) (Fig. 1A and B). The 6 B cell epitopes with different lengths of PRRSV M protein were predicted on the Immune Epitope Database (IEDB) (Table S2). According to the predicted B cell epitopes’ propensity and physicochemical properties, two peptides were selected, located at aa110-129 (LAPAHHVESAAGFHPITASD) and aa148-174 (VPGLKSLVLGGRRAVKRGVVNLVKYVK) of M protein (Fig. 1A, C and D). Interestingly, the B-cell linear epitopes contained in the two peptides was existing in sub-lineage 8.7, sub-lineage 1 and sub-lineage 5 PRRSV.

### Optimization of the peptide ELISA

**Fig. 2:**
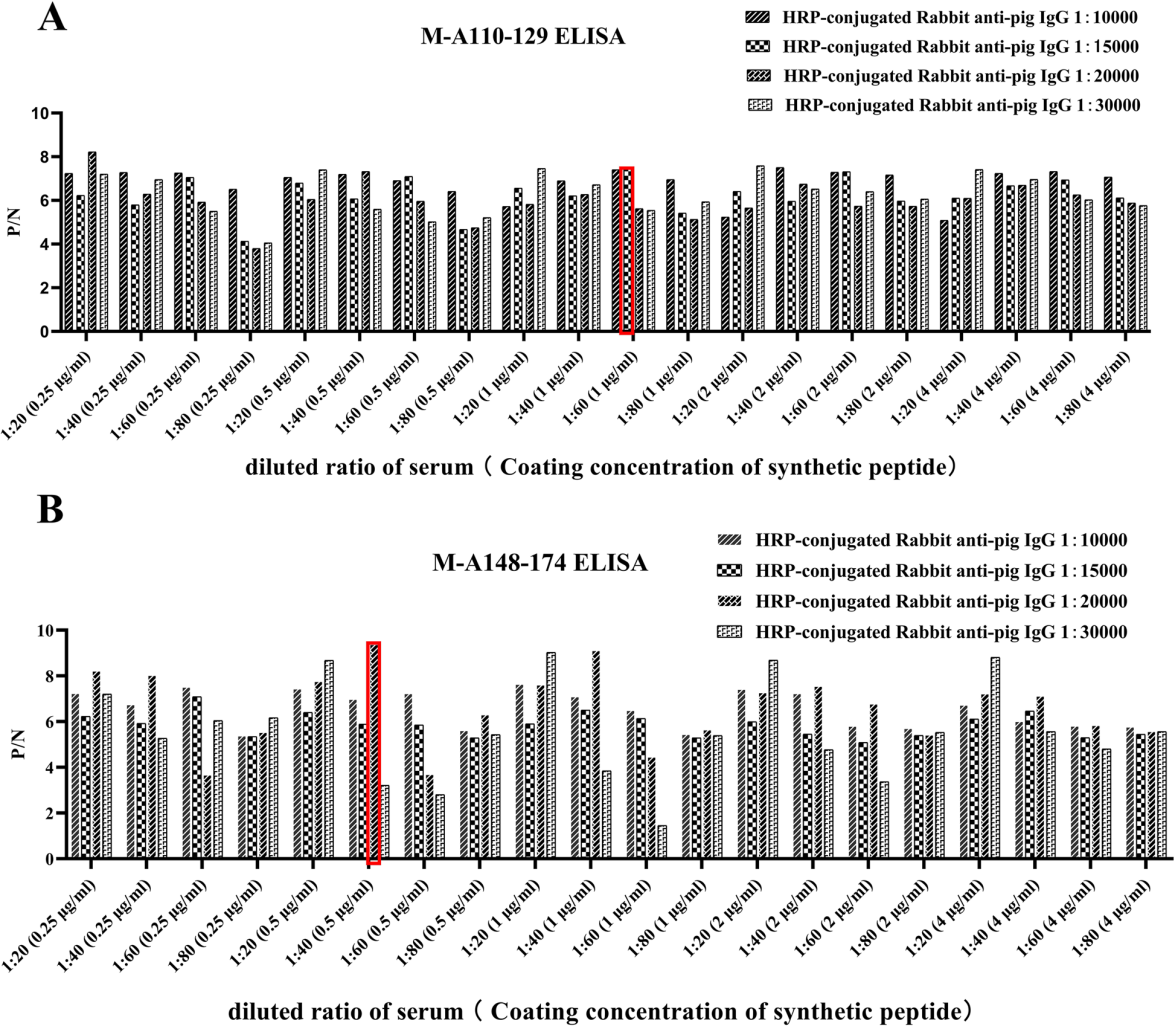
Optimization of peptide ELISA. **A** The optimization of M-A110-129 ELISA. **B** The optimization of M-A148-174 ELISA

Conditions for two peptide ELISA assays (M-A110-129 ELISA and M-A148-174 ELISA) were standardized by checkerboard titrations. The highest P/N value of the OD450nm ratio between positive and negative sera was the final condition of ELISA. The concentration of peptide coating, serum dilution and HRP conjugated antibody in M-A110-129 ELISA were 1 μg/ml, 1:60 and 1:15000, respectively (Fig. 2A). The concentrations of the peptide coating, serum dilution and HRP conjugated antibody in M-A148-174 ELISA were 0.5 μg/ml, 1:40 and 1:20000, respectively (Fig. 2B).

### Cutoff value, sensitivity, specificity of the peptide ELISA

PRRSV antibodies positivity serum samples from pigs collected in 2019, 2020 from pigs naturally infected with PRRSV and immunized with PRRSV vaccine. The negative serum samples from a PRRSV-free pig farm. All of the serum were further verified by IFA to confirm positivity or negativity for anti-PRRSV antibodies (Supplementary Fig. 1). These validated samples were used to determine the cutoff value of the peptide ELISA. An optimized cutoff value that maximized assay efficiency was demonstrated for an OD450nm ratio of 0.332 for M-A110-129 ELISA and an OD450nm ratio of 0.237 for M-A148-174 ELISA (Table S3).

**Fig. 3:**
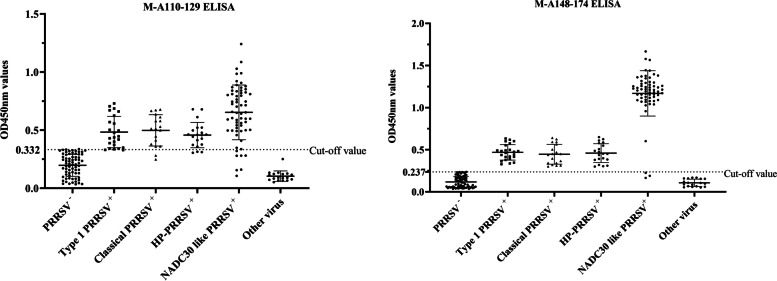
Specificity, sensitivity, and cross-reactivity of M-A110-129 ELISA (**A**) and M-A148-174 ELISA (**B**). The thin horizontal line represents the cut-of value (M-A110-129 ELISA: 0.332; M-A148-174 ELISA: 0.237). Statistically significant differences were compared between different subtypes PRRSV-positive serum and the other serum samples, including classical swine fever virus (CSFV)-positive, Porcine circovirus 2 (PCV2)-positive, foot-and-mouth disease virus (FMDV)-positive, pseudorabies virus-positive and PRRSV-negative serum samples

The anti-PRRSV positive sera collected from pigs infected with type 1 PRRSV, classical PRRSV, HP-PRRSV and NADC30 like PRRSV were detected by peptide ELISA. The M-A110-129 ELISA and M-A148-174 ELISA could detect antibodies against type 1 PRRSV, classical PRRSV, HP-PRRSV and NADC30 like PRRSV (Fig. 3). The two peptide ELISA assays in this study have wide applicability and can cross react with the specific antibodies of many subtypes PRRSV. The cross-reaction of the peptide ELISA was evaluated by testing the reactivity of sera raised against CSFV, PCV2, FMDV and PRV. As shown in Fig. 3, no cross-reaction between the NADC30-like PRRSV M peptide antigen and these sera was detected (Fig. 3).

**Fig. 4:**
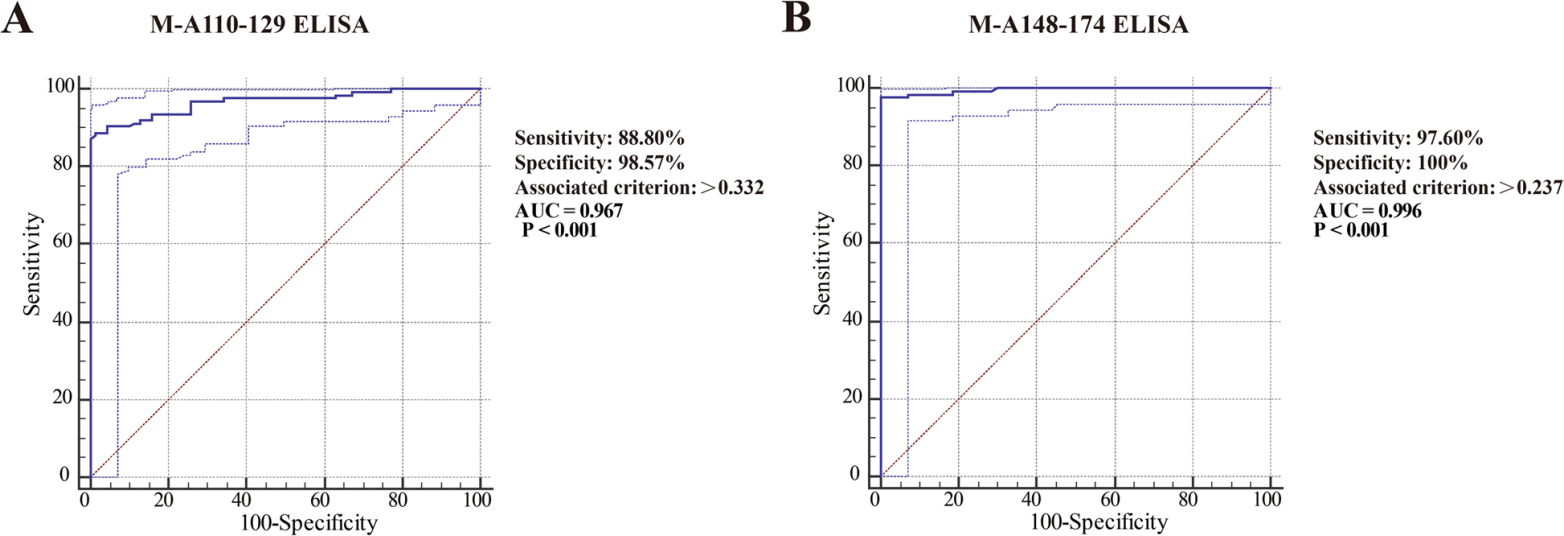
The receiver operating characteristic (ROC) curves of the M-A110-129 ELISA (**A**) and M-A148-174 ELISA (**B**) for the detection of antibodies against PRRSV. The ordinate represents the sensitivity of the peptide ELISA. The abscissa represents 1-specifcity of the peptide ELISA

The sensitivity of peptide ELISA is determined by the detection results of PRRSV antibody positive serum. The specificities of peptide ELISA are determined by the detection results of PRRSV antibody negative serum. As shown Fig. 3 and Table S3, both the sensitivities and specificities of M-A148-174 ELISA (97.6 and 100%) were higher than those of the M-A110-129 ELISA (88.8 and 97.60%). Comparison of ROC curves between the M-A110-129 ELISA and the M-A148-174 ELISA indicated that the specificity of the M-A110-129 ELISA was comparable to that of the M-A148-174 ELISA (*p* = 0.0174) (Table 1). Comparison of the AUC of peptide ELISA indicating that the M-A148-174 (0.996) ELISA had better accuracy to detect specific antibodies against PRRSV than the M-A110-129 ELISA (0.967) (Fig. 4).

**Table 1:** Comparison of ROC curves between the M-A110-129 ELISA and the M-A148-174 ELISA

Characteristics	M-A110-129-ELISA vs. M-A148-174-ELISA
Difference between areas	0.0282
Standard Error	0.0119
95% Confidence Interval	0.00505 to 0.0518
z statistic	2.384
Significance level	*P* = 0.0174

### The peptide ELISA effectively detects antibody responses in pigs

**Fig. 5:**
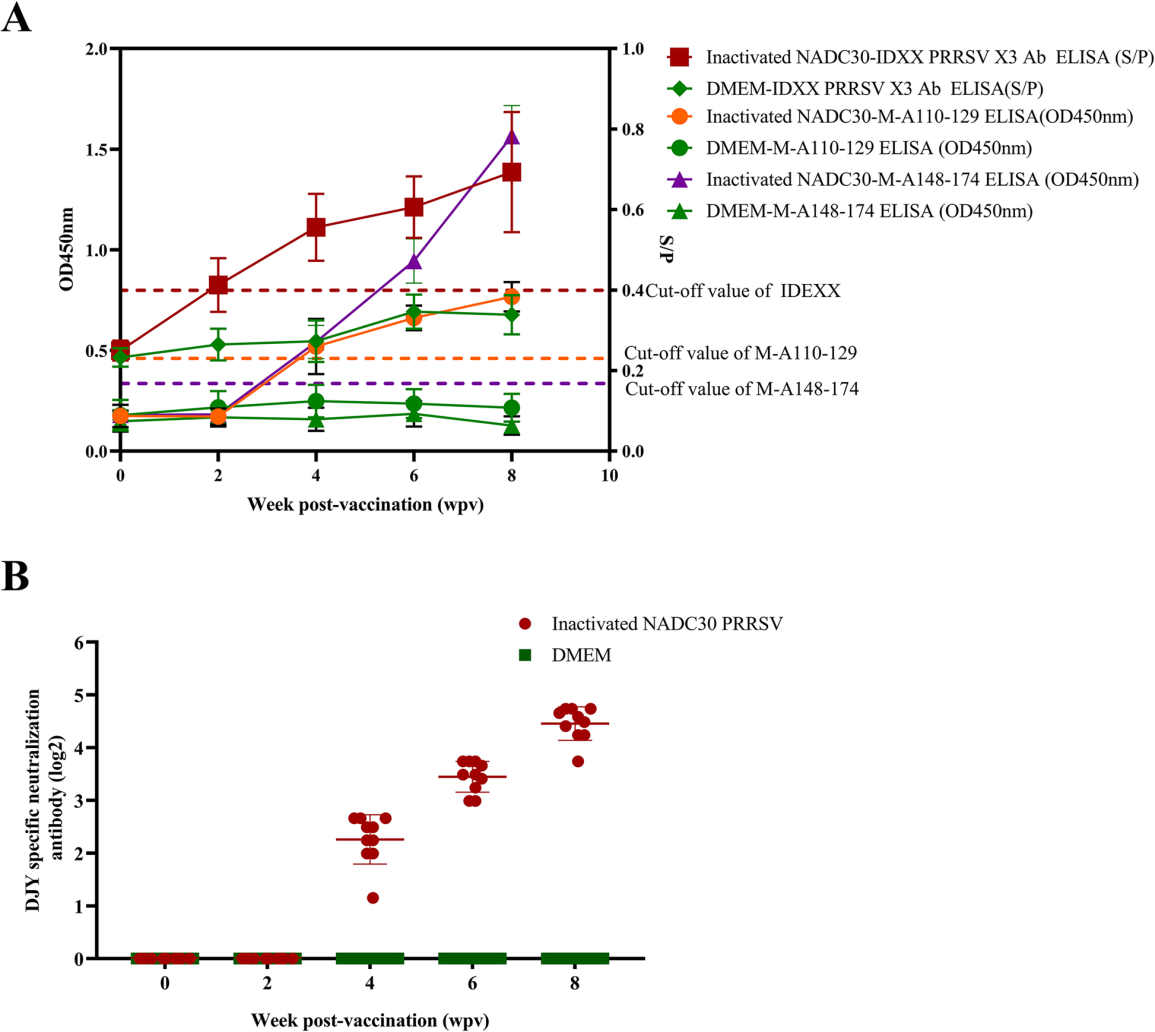
VN titers and ELISA results for sera from pigs immunized with NADC30-like PRRSV inactivated vaccine. **A** ELISA detected the serum samples collected at 0, 2, 3, 4, 6 and 8 weeks after inoculation. The red horizontal dotted line indicates the cutoff of the IDEXX PRRSV X3 Ab ELISA. The orange yellow horizontal dotted line indicates the cutoff of the M-A110-129 ELISA. The purple horizontal dotted line indicates the cutoff of the M-A148-174 ELISA. **B** Detection of neutralizing antibodies in the serum of pigs immunized with inactivated PRRSV vaccine at different time points

The IDEXX PRRSV X3 Ab ELISA, M-A110-129 ELISA, M-A148-174 ELISA and VN test were used to evaluate the immune response elicited by the NADC30-like PRRSV inactivated vaccine. The immunized group was inoculated with inactivated PRRSV vaccine, and the control group was not given treatment. The sera were collected at different time points post-vaccination and tested by ELISA and the VN test to measure PRRSV-specific IgG antibody and neutralizing antibody production. Specific IgG antibodies were detected in serum samples collected at different weeks post-vaccination pigs using the IDEXX PRRSV X3 Ab ELISA, M-A110-129 ELISA and M-A148-174 ELISA. The results of PRRSV specific antibodies in serum are shown in Table 2 and Fig. 5. The IDEXX PRRSV X3 Ab ELISA and M-A148-174 ELISA detected PRRSV specific antibodies in swine serum at 2 weeks after vaccine immunization, with positive rates of 80 and 10%, respectively. The M-A110-129 ELISA has poor applicability for the detection of PRRSV specific antibodies in pig serum after vaccination with PRRSV inactivated vaccine. The detection results of the first 3 weeks after vaccine immunization were judged to be negative by M-A110-129 ELISA. The VN titers were detected in swine serum at 4 weeks after vaccination, and then increased continuously. There were no PRRSV neutralizing antibodies or specific IgG in the serum of all control pigs. This result showed that the two peptide ELISA assays detected antibodies produced after vaccination in all pigs, but the results for M-A148-174 ELISA were significantly better than those for M-A110-129 ELISA.

**Table 2:** Comparison of peptide ELISA, IDEXX PRRSV X3 Ab ELISA, and VN test in detecting PRRSV antibody response in pigs

Antibody detection method	Week post-vaccination (wpv) (positive number/total number)									
	0		2		4		6		8	
	Vaccination^a^	Control^b^	Vaccination	Control	Vaccination	Control	Vaccination	Control	Vaccination	Control
M-A110-129 ELISA	0/10	0/10	0/10	0/10	7/10	0/10	10/10	0/10	10/10	0/10
M-A148-174 ELISA	0/10	0/10	1/10	0/10	10/10	0/10	10/10	0/10	10/10	0/10
IDEXX PRRSV X3 Ab ELISA	0/10	0/10	8/10	0/10	10/10	0/10	10/10	0/10	10/10	0/10
VN test	0/1	0/10	0/10	0/10	10/10	0/10	10/10	0/10	10/10	0/10

^a^Vaccination group was vaccinated with NADC30 like PRRSV inactivated vaccine, and sera were collected on indicated days and tested bypeptide ELISA, IDEXX PRRSV X3 Ab ELISA, and VN test

^b^Control group was not treated, and sera were collected on indicated days and tested by peptide ELISA, IDEXX PRRSV X3 Ab ELISA, and VN test

### Concordance between the VN test, IDEXX PRRSV X3 Ab ELISA and peptide ELISA

Serum samples were collected from 147 pigs immunized with NADC30-like PRRSV inactivated vaccine after 3 weeks. The sera were used to evaluate the consistency of the peptide ELISA with the IDEXX ELISA and VN detection. The results of the peptide ELISA and their agreement/disagreement with the IDEXX PRRSV X3 Ab ELISA and VN test in terms of seropositivity are summarized in Table 3. The results showed that the positive rates from high to low were IDEXX PRRSV X3 Ab ELISA, M-A148-174 ELISA, VN test and M-A110-129 ELISA. Cohen’s kappa coefficient analysis the consistency between ELISA assays and VN test. The results showed that the M-A148-174 ELISA had the best compatibility with VN test among the three ELISA assays, and the M-A148-174 ELISA had better compatibility with IDEXX PRRSV X3 Ab ELISA between the two peptide ELISA assays. The M-A148-174 ELISA results was in almost perfect agreement with the VN test, with a Cohen’s kappa coefficient of 0.8772 (95% confidence interval, 0.8710 to 0.8834). The M-A148-174 ELISA showed substantial agreement with the IDEXX PRRSV X3 Ab ELISA, with a kappa coefficient value of 0.7138 (95% confidence interval, 0.7216 to 0.7634).

**Table 3:** Concordance between the VN test, DEXX PRRSV X3 Ab ELISA, M-A109-129 ELISA, M-A148-174 ELISA

	M-A109-129 ELISA			M-A148-174 ELISA			IDEXX PRRSV X3 Ab ELISA		
	+	–	Total	+	–	Total	+	–	Total
VN									
+	50	13	63	63	0	63	63	0	63
–	2	82	84	9	75	84	28	56	84
Total	52	95	147	72	75	147	91	56	147
kappa coefficient^a^ (95% CI)	0.7870 (0.7791-0.7949)			0.8772 (0.8710-0.8834)			0.6316 (0.6217-0.6453)		
IDEXX PRRSV X3 Ab ELISA									
+	48	43	91	72	19	91			
–	4	52	56	0	56	56			
Total	52	95	147	74	73	147			
kappa coefficient^a^ (95% CI)	0.4020 (0.3972-0.4125)			0.7430 (0.7216-0.7634)					

^a^The significance of kappa coefficient value. < 0, no agreement; 0 to 0.20, slight agreement; 0.21 to 0.40, fair agreement; 0.41 to 0.60, moderate agreement; 0.61 to 0.80, substantial agreement; and 0.81 to 1.0, almost perfect agreement

### Evaluation of PRRSV-specific antibodies in field serum samples using the peptide ELISA

The M-A110-129 ELISA, M-A148-174 ELISA and Hipra PRRSV ELISA kit were evaluated for detection of PRRSV-specific antibodies in sera collected from farmed pigs infected with unknown field PRRSV strains. A total of 260 IDEXX PRRSV X3 Ab ELISA-positive field serum samples were further tested by Hipra PRRSV ELISA, two peptide ELISA along with IFA verification (Supplementary Fig. 2). Among these field samples, two IDEXX PRRSV X3 Ab ELISA-positive serum samples were found to be negative using both the IFA and peptide ELISA (both M-A110-129 ELISA and M-A148-174 ELISA), suggesting two false-positive results for this sample based on the IDEXX PRRSV X3 Ab ELISA. Compared with the detection results of IFA, the concordance rates of the Hipra PRRSV ELISA kit, M-A110-129 ELISA, and M-A148-174 ELISA in the field seropositive detection results were 91.08, 86.32, and 95.35%, respectively (Table 4). Among the unexpected false-negative results with the M-A110-129 ELISA and M-A148-174 ELISA, 12 false-negative serum samples of the M-A148-174 ELISA were associated with the same serum of the M-A110-129 ELISA. The coincidence rate of the M-A148-174 ELISA was the highest among the 3 ELISA assays with sacsin or sacsin polypeptide epitopes as coating antigens. Although false-positive results exist for the IDEXX ELISA method, the features of high sensitivity make it irreplaceable for field serodiagnosis of wild-type virus infection.

**Table 4:** Comparison of field samples detected by peptide ELISA, Hipra PRRSV ELISA kit, IDEXX PRRSV X3 Ab ELISA and IFA

Serum Group	No. of seropositive detected by analysis/total No. of tested serum samples			
	M-A110-129 ELISA	M-A148-174 ELISA	Hipra PRRSV ELISA kit	IFA
IDEXX PRRSV X3 Ab ELISA-positive	221/260	246/260	235/260	258/260

## Discussion

ELISA has been used in PRRSV serological tests for its feasibility, sensitivity, rapidity and being suitable for large-scale use. At present, there are many studies on the specificity and sensitivity of commercial PRRSV ELISA kits [25–28]. The IDEXX PRRSV Ab ELISA kit and LSI PRRSV-Ab ELISA kit are two commonly used PRRSV antibody detection kits [29]. However, the high price of these kits is an important limitation. And the results of the two kits were in poor agreement with the VN test [30]. In this study, two peptides covering B cell epitopes based on the NADC30-like PRRSV M protein were screened by bioinformatics analysis to establish peptide ELISA assays for the detection of PRRSV specific antibodies. The M protein is the most conserved in all proteins of PRRSV, and its B cell linear epitopes have been studied in detail. At present, five B-cell linear epitopes of M protein have been reported, which are located in aa3-6, aa133-159, aa151-165, aa155-163 and aa161-174 of the M protein [14, 31, 32]. The amino acid comparison between HP-PRRSV and NADC30 PRRSV M protein showed that the aa159 R-S, aa160 K-R and aa164 Q-R mutations have occurred in aa155-163 and aa161-174 B-cell linear epitopes, but it is still very stable. Four α-helix structures were predicted in the M protein and did not overlap with the reported B cell linear epitopes and two peptide sequences selected in this study (Fig. 1A).

ELISA as antibody detection technology plays an important role in PRRSV epidemic monitoring and vaccine immune evaluation [26]. Commercial ELISA kits have high sensitivity and specificity to detect specific antibodies against PRRSV, but they have poor agreement with the VN test to evaluate the immune effect of the vaccine [30]. In this study, two peptide ELISA assays were established and evaluated their sensitivity, specificity, consistency with VN and clinical serodiagnostic ability. Compared to the M-A110-129 ELISA, the M-A148-174 ELISA had a stronger positive value than the M-A110-129 ELISA, and it displayed better diagnostic efficiency, with higher values for sensitivity, specificity and AUC (M-A110-129 ELISA vs. M-A148-174 ELISA: 88.8% vs. 97.6, 98.57% vs. 100%, and 0.967 vs. 0.996, respectively). In the M-A148-174 ELISA, the antibody against PRRSV in the serum of pigs could be detected on as early as 2 weeks post-vaccination, but the detection rate was lower than that of the IDEXX ELISA kit. The M-A148-174 ELISA had better consistency with VN detection (kappa coefficient value of M-A148-174 ELISA vs. the kappa coefficient value of M-A110-129 ELISA: 0.8772 vs. 0.7870). As the two ELISA kits and two peptide ELISA assays were measuring antibodies to different antigens, significant variation would not be unexpected. The IDEXX ELISA kit, coating with N protein of PRRSV, which was the most immunogenic and stable of all structural and nonstructural proteins of PRRSV. The Hipra ELISA kit, coating with GP2, GP3, GP4, GP5 and M proteins of PRRSV. The GP2, GP3, GP4 and GP5 proteins had poor homology in different subtypes of PRRSV. Two peptide ELISA assays in this study, coating with different peptides, which were located in M protein and contained identified B cell linear epitopes. In addition, the detection results of PRRSV-specific antibody in field serum showed that the M-A148-174 ELISA (95.35%) had more sensitivity than the M-A110-129 ELISA (86.32%) and Hipra PRRSV ELISA kit (91.08%). This may be related to the inclusion of two B cell linear epitopes in the peptide antigen of M-A148-174 ELISA and higher purity of synthetic peptide.

## Conclusion

In summary, we have developed two peptide ELISA assays with two synthetic M peptides, which could detect antibodies against NADC30-like PRRSV. Comparing the sensitivity and specificity of the two peptide ELISA assays, the M-A148-174 ELISA can be considered to be more suitable for serodiagnosis of PRRSV infection in pigs. The results of the M-A148-174 ELISA were in perfect agreement with the VN test, so it may be used for the evaluation of the inactivated NADC30-like PRRSV vaccine.

### Materials and assays

#### Virus, cells, PRRSV-specific serum and reagents

PRRSV DJY strain (type 2 NADC30-like PRRSV), which was isolated and identified by our lab (GenBank accession no.MT075480) [33], was used in the present work. The virus was grown and titrated in MARC-145 cells. MARC-145 cells were propagated in Dulbecco’s modified Eagle’s medium (DMEM, GIBCO, USA) supplemented with 10% fetal bovine serum (FBS) (GIBCO, USA). The positive serum against PRRSV DJY strain was stored in our Lab. TMB Chromogen Solution for ELISA (P0209) and Stop Solution for TMB Substrate (450 nm, sulfuric acid-free) (P0215) were purchased from Beyotime. HRP-conjugated rabbit anti-pig IgG (D111051) was purchased from Angon Biotech (Shanghai) Co., Ltd.

#### PRRSV inactivated vaccine preparation and vaccination

NADC30-like PRRSV DJY was chemically inactivated using binary ethyleneimine (BEI). 10^7.0^ TCID50/mL virions were obtained from MARC-145 cells. After freezing and thawing and centrifuging at 4000 rpm for 15 mins, the supernatant was collected and then inactivated by the addition of 1 M BEI to a final concentration of 2 mM at 37 °C overnight. The remaining BEI was neutralized by the addition of 20% sodium thiosulfate. Virus inactivation was verified by the absence of viral growth in MARC-145 cell cultures. The inactivated vaccine against PRRSV was formulated with 20% Montanide™ GEL01 adjuvant (Seppic, Paris, France).

Four-week-old growing pigs (*n* = 157) were purchased from a PRRSV-free pig farm in Sichuan Mianyang. All pigs were verified as serologically negative for PRRSV by the IDEXX PRRSV X3 Ab ELISA kit. Pigs (*n* = 147) were vaccinated with the NADC30-like PRRSV inactivated vaccine by intramuscular injection. The control group (*n* = 10) was not treated. Serum samples for were collected at 0, 2, 3, 4, 6 and 8 weeks post-vaccination (wpv) for ELISA and neutralizing antibody detection. After the experiment, all pigs were raised normally and not killed.

#### Virus neutralization test

Serum-neutralizing antibody titers were determined by a virus neutralization (VN) test. The serum samples were diluted two-fold (1:4 to 1:128) and mixed with an equal volume of 100 TCID_50_/0.1 mL PRRSV DJY diluent for 1 h at 37 °C in 5% CO_2_. The treated virus was added to a 96-well plate with MARC-145 cells for 1 h at 37 °C in 5% CO_2_. Then, 100 μL of DMEM containing 2% FBS was added into wells. After incubation for 48 h, the neutralization titers were calculated by counting the PRRSV-specific cytopathic effect (CPE) of each well.

#### Immunofluorescence assay (IFA)

Monolayers of MARC-145 cells grown in 96-well plates (Corning Inc.) and infected with PRRSV DJY at 37 °C in 5% CO_2_ atmosphere for 48 h, Cells were then fixed with 4% paraformaldehyde (Beyotime, Shanghai, China), permeabilized with PBS-T buffer and blocked with 1% BSA. Then, 50 μL of diluted serum samples (1:100) were added to paired wells (for PRRSV-infected versus uninfected MARC-145 cells) and incubated for 1 h. After three washes, goat anti-pig IgG H&L (FITC) (Abcam, USA) was used to detect specific antibody binding. Fluorescence results were observed and imaged with a Nikon TE300 fluorescent microscope.

#### B cell linear epitope prediction of M protein and synthetic peptide

The ORF6 gene sequences of NADC30-like PRRSV and HP-PRRSV were aligned by Mega (Table S1). The Immune Epitope Database and analysis resource (IEDB)(iedb.org/)was used to predict B cell epitopes of the NADC30-like PRRSV M protein. The 3D structure of the M protein was predicted and simulated using SWISS-MODEL (https://swissmodel.expasy.org/) and the models were visualized in PyMOL (https://pymol.org). The peptides were synthesized by Angon Biotechnology (Shanghai) Co., Ltd.

#### Elisa

The 96-well plate (Corning; Corstar® assay clear, Flat bottom polystyrene plate, USA) was coated with 100 μL synthetic peptide (0.25, 0.5, 1, 2, 4 μg/mL) in 0.05 M carbonate and bicarbonate buffer (pH 9.6), and was incubated at 4 °C overnight for adsorption. The antigen solution was discarded and washed three times with PBS containing 0.05% Tween 20 (PBST). The wells were treated with 150 μL PBS buffer containing 1% skim milk at 37 °C for 1 h. After that, the plate was washed three times with PBST. The serum samples (negative and positive sera) were diluted with antibody diluent, added 100 μL/well and incubated at 37 °C for 1 h, washed with PBST three times. HRP-conjugated rabbit anti-pig IgG was diluted with antibody diluent, added at 100 μL/well and incubated at 37 °C for 1 h, and washed with PBST three times. Then, 50 μL of tetramethyl benzidine dihydrochloride (TMB) was added into the wells; after 10 mins, the substrate-chromogen reaction was terminated with the addition of 50 μL of 2 M H_2_SO_4_ per well. The optical density (OD) value was determined at 450 nm by a microplate reader. The dilution ratio of test serum and HRP-conjugated antibody and optimal concentration of synthetic peptide were determined by the checkerboard titration method as described earlier [34].

#### Cutoff determination, diagnostic sensitivity and diagnostic specificity

To determine the sensitivity and specificity of the peptide ELISA (M-A110-129 ELISA, M-A148-174 ELISA), 195 serum samples from either PRRSV-infected pigs or non-PRRSV-infected pigs were tested. The 63 anti-PRRSV antibody-positive samples collected in 2019 from pigs naturally infected with NADC30-like PRRSV strain DJY were used as positive controls [33]. The 24 anti-type 1-PRRSV antibody-positive samples collected in 2020 from pigs naturally infected with type 1 PRRSV strain SC2020-1 were used as positive controls [35]. The 20 anti-HP-PRRSV antibody-positive samples collected in 2020 from pigs immunized with JXA1-R vaccine and 18 anti-classical-PRRSV antibody-positive samples collected in 2020 from pigs immunized with CH-1R vaccine were used as positive controls. Moreover, 70 negative serum samples from a PRRSV-free pig farm in Sichuan Mianyang were included as negative controls. All anti-PRRSV antibody-positive serum samples or negative samples were further confirmed by IFA of PRRSV-infected MARC-145 cells. Receiver operating characteristic (ROC) analysis assessments were performed using MedCalc (Version 19.0.7, MedCalc Software, Belgium) [36].

#### Concordance between the VN test, IDEXX PRRSV X3 Ab ELISA and peptide ELISA

Sera collected from pigs (*n* = 147) immunized with PRRSV inactivated vaccine for 3 weeks were tested using the VN test, IDEXX PRRSV X3 Ab ELISA and peptide ELISA. The results were analyzed to compare the VN test, peptide ELISA and IDEXX PRRSV X3 Ab ELISA. The statistical significance was evaluated by Fisher’s exact test. Cohen’s kappa coefficient value was calculated according to the guidelines of Landis and Koch [37].

#### Evaluation of field serum samples

A total of 260 PRRSV-positive (as defined by IDEXX PRRSV X3 Ab ELISA) field pig serum samples collected from pigs on various farms (supplied by Sichuan Chiyang Agricultural Development Co., Ltd.) were further tested by both peptide ELISA and Hipra PRRSV ELISA kit with IFA verification.

## Supplementary Information

**Additional file 1: Supplementary Table 1.** The sequences of the PRRSV strains available in NCBI data. **Supplementary Table 2.** Prediction of B cell linear epitopes in IEDB. **Supplementary Table 3.** ROC Analysis for M-A110-129-ELISA and M-A148-174-ELISA. **Supplementary Figure 1.** Immunofluorescence analysis identification of serum for peptide ELISA optimization. MARC-145 cells were infected with PRRSV for 48 h. **Supplementary Figure 2.** Immunofluorescence analysis identification of the field serum samples for evaluation the peptide ELISA. MARC-145 cells were infected with PRRSV for 48 h.

## Data Availability

All data generated during this study are publicly available. This data can be found at: https://www.ncbi.nlm.nih.gov/nuccore/MT075480.1. However, the raw data is available from the corresponding author upon reasonable request.

## Abbreviations

PRRSV: Porcine reproductive and respiratory syndrome virus
ROC: Receiver operating characteristic curve
AUC: Area Under the Curve
ELISA: Enzyme linked immunosorbent assay
ORFs: Open reading frames
SARS-CoV-2: Severe acute respiratory syndrome coronavirus 2
GETV: Getah virus
IBV: Infectious bronchitis virus
ILT: Avian infectious laryngotracheitis virus
IEDB: Immune Epitope Database
OD450nm: Optical density 450
CSFV: Classical swine fever virus
PCV2: Porcine circovirus 2
PRV: Pseudorabies virus
FMDV: Foot-and-mouth disease virus
IgG: Immunoglobulin G
VN: Virus neutralizing antibody
TCID50: 50% tissue culture infective dose
DMEM: Dulbecco’s modified Eagle’s medium
MARC-145: African green monkey embryonic kidney epithelial Cells
HRP: Horseradish peroxidase
CPE: Cytopathic effect
FITC: Fluorescein isothiocyanate
TMB: Tetramethyl benzidine dihydrochloride
PBST: 0.5% Tween-20 in PBS
IFA: Immunofluorescence assay
