# Supplementary material for: Establishment of a peptide-based enzyme-linked immunosorbent assay for detecting antibodies against PRRSV M protein
Source: BMC Vet Res. 2021 Nov 19;17:355. doi: 10.1186/s12917-021-03060-z (PMC8602981; doi:10.1186/s12917-021-03060-z)
Supplement: Supplementary file 1 — Additional file 1: Supplementary Table 1. The sequences of the PRRSV strains available in NCBI data. Supplementary Table 2. Prediction of B cell linear epitopes in IEDB. Supplementary Table 3. ROC Analysis for M-A110-129-ELISA and M-A148-174-ELISA. Supplementary Figure 1. Immunofluorescence analysis identification of serum for peptide ELISA optimization. MARC-145 cells were infected with PRRSV for 48 h. Supplementary Figure 2. Immunofluorescence analysis identification of the field serum samples for evaluation the peptide ELISA. MARC-145 cells were infected with PRRSV for 48 h. [file 12917_2021_3060_MOESM1_ESM.docx]

**Supplementary Table 1.** The sequences of the PRRSV strains available in NCBI data.

| Strain | GenBank Accession | Country | Isolate Date | Strain | GenBank Accession | Country | Isolate Date |
| --- | --- | --- | --- | --- | --- | --- | --- |
| NADC30 | JN654459 | USA | 2012 | FZ16A | KY761966 | China | 2016 |
| XW004 | KF724397 | China | 2013 | SCnj16 | MF196906 | China | 2016 |
| MN6-CHina | KP283401 | China | 2014 | VR2332 | EF536003 | China | 2007 |
| Minnesota16 | KP283404 | USA | 2014 | DK-2010-10-7-1 | KC862580 | China | 2010 |
| TJnh1501 | KX510269 | China | 2015 | GS2004 | EU880443 | China | 2004 |
| SH/CH/2016 | KY495781 | China | 2016 | HK14 | KF287141 | China | 2013 |
| FJXS15 | KX758250 | China | 2015 | A2MC2 | KX462792 | USA | 2016 |
| CH-1R | EU807840 | China | 2008 | HPBEDV | EU236259 | China | 2007 |
| HuN4 | EF635006 | China | 2007 | HN2007 | EU880437 | China | 2009 |
| SDSU73 | JN654458 | USA | 2011 | JXA1-R | FJ548853 | China | 2009 |
| TJ | EU860248 | China | 2008 | SC2012 | KM189443 | China | 2012 |
| SHH | EU106888 | China | 2007 | 15SC1 | KX815426 | China | 2015 |
| DJY | MT075480 | China | 2019 |  |  |  |  |

**Supplementary table 2.** Prediction of B cell linear epitopes in IEDB.

| **PRRSV strain** | **No.** | **Start** | **End** | **Peptide** | **Length** | **PRRSV strain** | **No.** | **Start** | **End** | **Peptide** | **Length** |
| --- | --- | --- | --- | --- | --- | --- | --- | --- | --- | --- | --- |
| NADC30 like PRRSV(DJY) | 1 | 5 | 14 | IDDFCNDSTA | 10 | HP-PRRSV (JXA1) | 1 | 6 | 15 | DDFCNDSTAP | 10 |
|  | 2 | 39 | 39 | G | 1 |  | 2 | 39 | 39 | G | 1 |
|  | 3 | 90 | 90 | E | 1 |  | 3 | 66 | 68 | EST | 3 |
|  | 4 | 110 | 129 | LAPAHHVESAAGFHPITASD | 19 |  | 4 | 110 | 129 | LAPAHHVESAAGFHPIAAND | 20 |
|  | 5 | 137 | 143 | RPGSTTV | 7 |  | 5 | 137 | 143 | RPGSTTV | 7 |
|  | 6 | 148 | 168 | VPGLKSLVLGGRRAVKRGVVN | 21 |  | 6 | 148 | 168 | VPGLKSLVLGGRKAVKQGVVN | 21 |

**Supplementary table 3.** ROC Analysis for M-A110-129-ELISA and M-A148-174-ELISA.

| **Characteristics** | **Value for M-A110-129 ELISA** | **Value for M-A148-174 ELISA** |
| --- | --- | --- |
| Optimized cutoff (OD450nm) | 0.332 | 0.237 |
| Diagnostic sensitivity (%) | 88.80 | 97.60 |
| 95% confidence interval | 78.4 - 95.3 | 86.7 - 99.4 |
| Diagnostic specificity (%) | 98.57 | 100 |
| 95% confidence interval | 92.3-100 | 94.9 - 100.0 |
| AUC | 0.967 | 0.996 |
| 95% confidence interval | 0.932-0.987 | 0.973-1.0 |
| Significance level P (Area=0.5) | ＜0.0001 | ＜0.0001 |


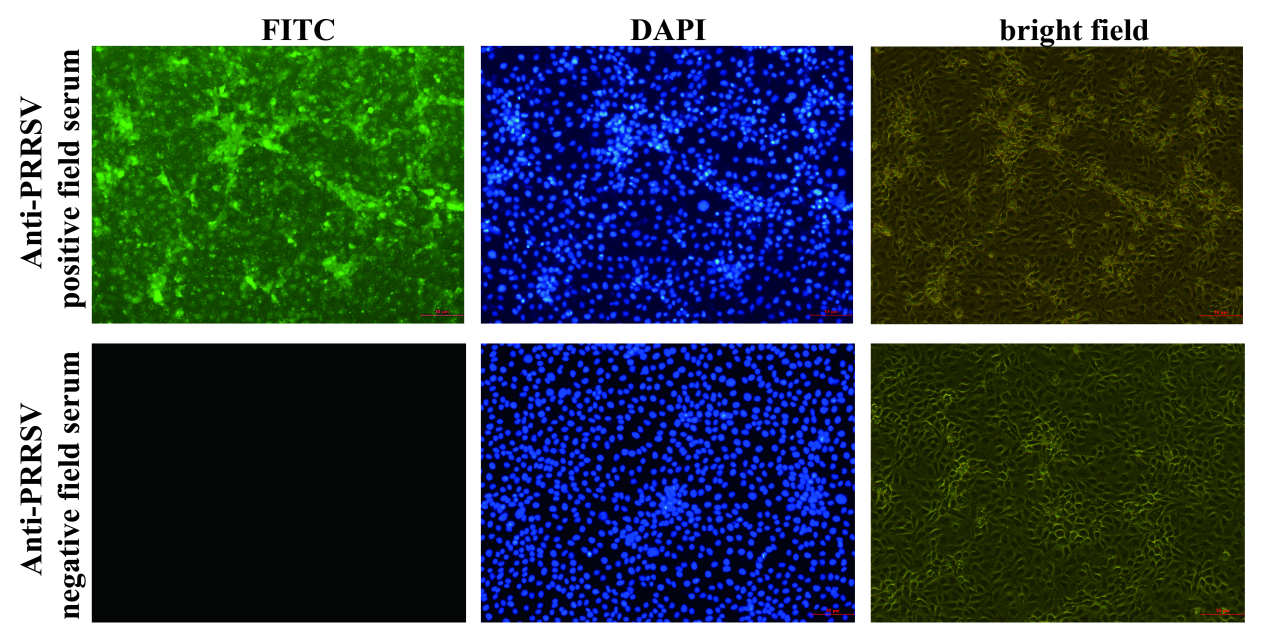


**Supplementary Figure 1.** Immunofluorescence analysis identification of serum for peptide ELISA optimization. MARC-145 cells were infected with PRRSV for 48h.


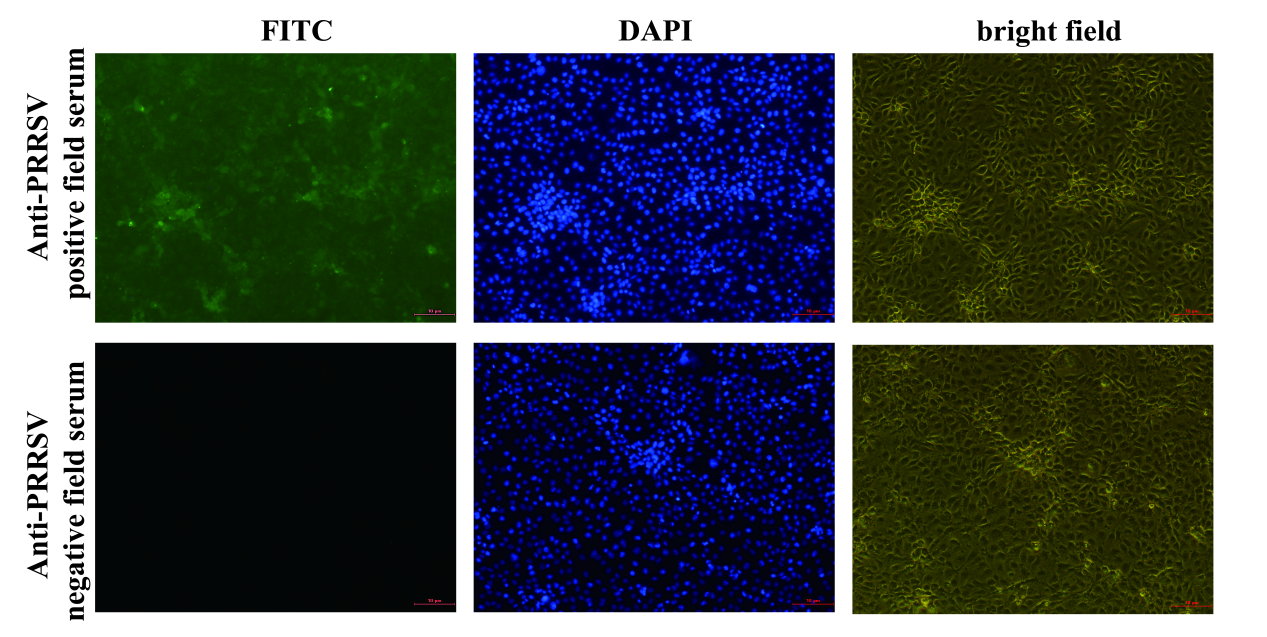


**Supplementary Figure 2.** Immunofluorescence analysis identification of the field serum samples for evaluation the peptide ELISA. MARC-145 cells were infected with PRRSV for 48h.
